# Peripheral Ameloblastoma of Upper Gingiva in a Patient with Port-Wine Stain

**DOI:** 10.1155/2020/2870715

**Published:** 2020-05-09

**Authors:** Natheer H. Al-Rawi, Sahar Othman, Ab Rani Samsudin

**Affiliations:** College of Dental Medicine, University of Sharjah, Sharjah, UAE

## Abstract

The peripheral ameloblastoma (PA), also known as extraosseous ameloblastoma, is a rare soft tissue tumor of odontogenic origin, accounting for 1–5% of all ameloblastoma. In some cases, saucerization of underlying bone is the only radiological evidence of this lesion, and PA has identical histological characteristics of intraosseous ameloblastoma. However, it is slow growing, less aggressive, and less invasive in nature. The present report describes a rare case of PA in the maxillary labial gingiva of a 37-year-old man with port-wine vascular malformation. PA was clinically diagnosed as a pyogenic granuloma, and following the surgical treatment of the lesion, its histological features were of ameloblastoma. This case illustrates the importance of including peripheral ameloblastoma in the differential diagnosis of painless exophytic gingival swelling.

## 1. Introduction

Ameloblastoma is by far the most common benign epithelial odontogenic tumor, occurring mainly in the jaw. According to the WHO, ameloblastoma is generally classified into four variants: solid/multicystic, desmoplastic, unicystic, and peripheral. The first three variants are of intraosseous type, and the fourth is extraosseous. The peripheral ameloblastoma (PA) is an uncommon extraosseous variant of ameloblastoma, and only less than 200 cases of PA are having proper recognition in the literature [1]. PA is clinically presented as a painless, firm, smooth exophytic growth with pedunculated or sessile base and a normal mucosal color [2]. The overall average age of occurrence for peripheral ameloblastoma is reported to be 52.1 years, which is significantly higher than its intraosseous counterpart, having a mean age of 37.4 years [3]. In addition, despite the fact that peripheral ameloblastoma is usually confined to the gingiva with no evidence of bone involvement, superficial bony erosion known as cupping or saucerization may be detected radiographically or at surgery.

Peripheral ameloblastoma is more commonly located in the mandible, especially the lingual gingiva in the premolar region, followed by the anterior region [4]. In the maxilla, however, the most common location is the soft palatal tissue of the tuberosity area [5,6]. This paper describes a patient with peripheral ameloblastoma, highlighting the clinical, radiological, and operative findings and histological features of this rare lesion involving the maxilla.

## 2. Case Presentation

**Figure 1:**
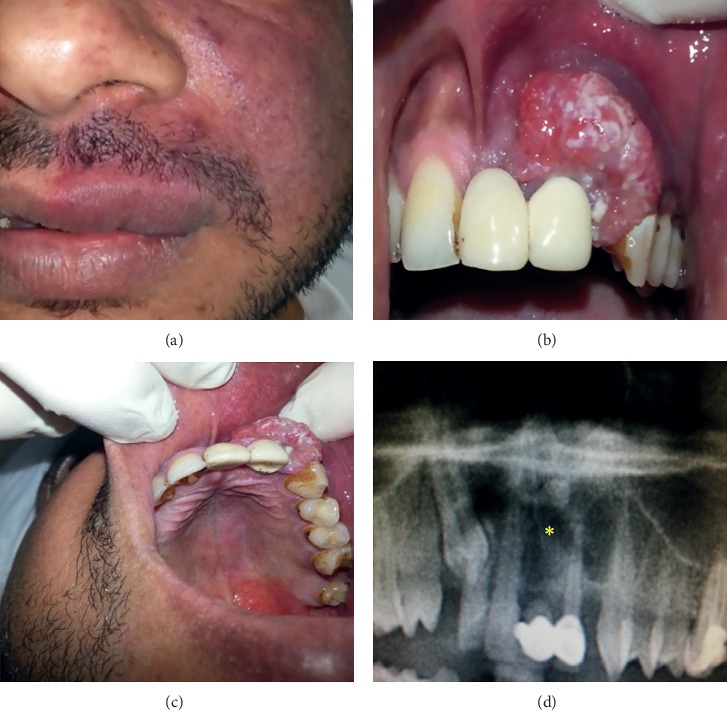
(a) Port-wine stain along the distribution of the maxillary nerve. (b) Clinical view: firm, pedunculated, nontender, and nonpulsatile growth. (c) Palatal view of the lesion. (d) Panoramic view: well-demarcated periapical radiolucency in the alveolar region.

A 37-year-old healthy man was referred to the University Dental Hospital of Sharjah for the management of a soft tissue mass on the left anterior part of maxillary gingiva, being there for a duration of six months. According to the patient, the swelling enlarged gradually during that period, with frequent episodes of recurrent bleeding without any history of trauma. The patient also presented with a congenital port-wine stain on the left facial skin along the distribution of the maxillary division of the trigeminal nerve (Figure 1(a)).

Intraoral clinical examination revealed a labial painless bluish red mass, about 3 × 4 cm in diameter, which was a firm, pedunculated, nontender, and nonpulsatile lesion, extending from the upper right central incisor to the upper left canine (Figure 1(b)).

The oral mucosa overlying the lesion showed some degree of keratinization with multiple superficial ulcerations that bleed easily on a slight touch. The neighboring upper left lateral incisor was nonvital, mobile, and crowned, whereas the upper left canine responded to the vitality test. Finally, the upper right central incisor was firm. The patient had poor oral hygiene and suffered from generalized chronic periodontitis. No regional lymphadenopathy was present, and the facial port-wine stain extended intraorally to involve the upper left labial mucosa, the buccal gingiva, and the whole left side of the hard and soft palates (Figure 1(c)).

A panoramic radiograph showed a well-demarcated periapical radiolucency in the alveolar region extending from the root of tooth number 11 till tooth number 23, with nonvital tooth number 22. A supernumerary tooth was detected distal to the root of tooth number 21 within the radiolucent lesion, and an impacted right maxillary canine was present, yet it was irrelevant to the lesion. The nasal cavity and maxillary sinus cavity appeared normal and free from the lesion (Figure 1(d)).

The clinical differential diagnosis included hemangioma, pyogenic granuloma, peripheral giant cell granuloma, and peripheral ossifying fibroma.

## 3. Pathological Findings

**Figure 2:**
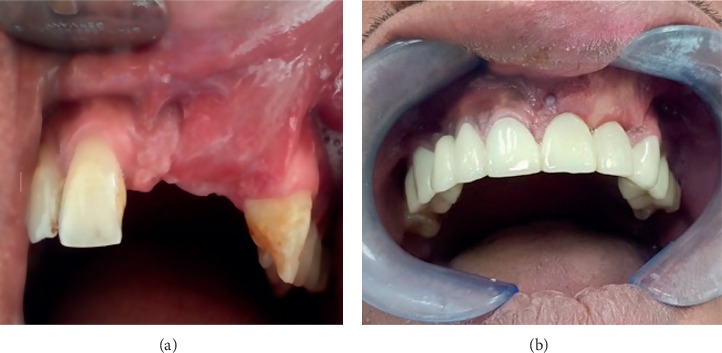
(a) Postoperative view (after 3 months). (b) Postoperative view (after 3 years).

A soft tissue incisional biopsy of the lesion was taken under local anesthesia, and then the specimen was sent for histopathological examination. A brisk bleeding episode was provoked, yet it was well controlled with gauze and finger pressure. H&E stained sections of the soft tissue component revealed islands of odontogenic epithelium, arranged in plexiform, as well as in the follicular pattern. The proliferating basaloid cells were organized in nests or islands, with several microcysts in the center. The nuclei of peripheral cells were located at the opposite pole of the basement membrane. These islands of the odontogenic epithelium were seen immediately underneath the epithelial layer in the lamina propria, and the histologic findings were consistent with features of peripheral ameloblastoma. The maxillofacial surgeon explained the treatment plan to the patient and addressed the need for a complete removal of the soft tissue lesion alongside the bone lesion, due to the high possibility of lesion extension to the radiolucent area that was previously detected on orthopantomogram (OPG). The patient refused excision under general anesthesia (GA) and preferred the removal of the soft tissue mass only. Then, based on clinical, radiological, and histological findings and the patient's preference, a decision was made to excise the lesion under local anesthesia. Wide excision of the tumor was done to achieve complete disease-free margins, and the underlying saucerized bone was curated, leaving a raw bleeding area. Hemostasis was achieved using bone wax, and wound closure was done using labial mucosa advancement flap. Sutures were removed on the tenth postoperative day, and a temporary denture was placed. The patient was on frequent review up to three years with no evidence of recurrence (Figures 2(a) and 2(b)).

**Figure 3:**
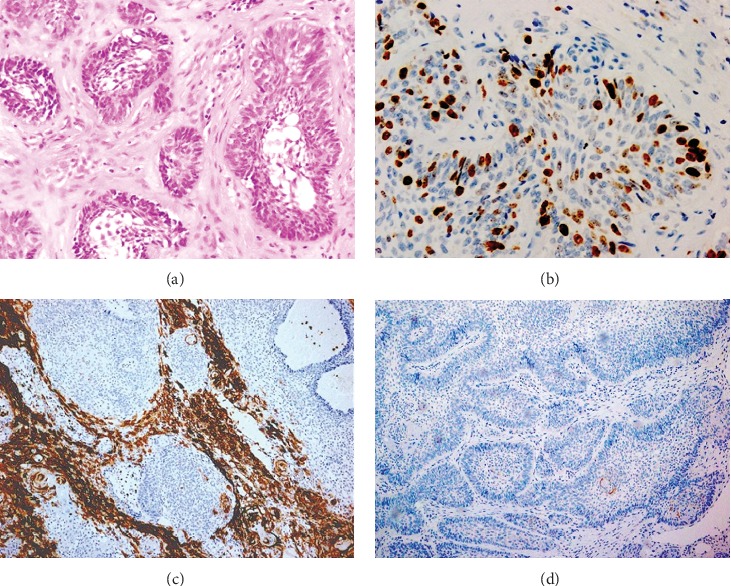
(a) Follicular epithelia with microcyst formation in the center (H&E staining, original magnification; 40×), (b) expression within nuclei of follicular epithelia (original magnification; 40×), (c) CD10 expression in stromal cells (original magnification; 10×), (d) Ber-EP4 negative expression (original magnification; 10×).

The whole mass was transferred to the histopathology lab for further evaluation. The superficial part of the lesion showed typical odontogenic epithelium with follicular and some plexiform patterns; however, the deepest portion of the lesion showed aggressive behavior of the tumor with marked hyper cellularity with basaloid components, resembling basal cell carcinoma (Figure 3(a)). Immunohistochemical stains were done to assess the aggressiveness of the lesion. Ki 67, CD10, and BER-EP4 were used. Ki67 was used to determine the proliferation rate of the tumor, which was very high (33% ± 7.85) (Figure 3(b)). CD10 was used to determine the stromal invasion of the tumor (Figure 3(c)), and Ber-EP4 was used to differentiate the lesion from basal cell carcinoma (Figure 3(d)). Stromal CD10 was scored according to the similar system used by Zu et al. [7]. The score of the present study was >50% of stromal cells positive with intense stain against anti-CD10 monoclonal antibody. Ber-EP4, which is a basal cell carcinoma marker, was negative for both stromal and follicular epithelial cells (Figure 3(d)). This confirmed the diagnosis of peripheral ameloblastoma.

## 4. Discussion

The first well-documented case of peripheral ameloblastoma is reported by Stanley and Krogh in 1959 [8]. Since then, very few cases have been reported [1,9]. It is commonly seen in men (65%) with an overall average age of 52.1 years at the time of diagnosis [2]. The patient reported in this case is 15 years younger than the average age of occurrence, and the lesion occurred in the anterior maxilla instead of the mandible, despite the literature reports that states the mandible as the most common site for peripheral ameloblastoma accounting for 70.9% of all cases [3]. Maxillary lesions are less common and comprise only 28% of all lesion with anterior gingiva as the preferred site [9,10]. Malignant PA has also been reported in the literature [11,12].

The potential source of PA includes the extraosseous remnant of dental lamina or basal cell layer of the surface epithelium. PA is typically present as a slow, painless, exophytic growth of gingiva or oral mucosa [13].

Generally, the color of peripheral ameloblastoma varies between pink and red or dark red and is usually present as a painless, sessile mass, which is usually a firm growth with a smooth surface, occasionally exhibiting a papillary or warty appearance [14]. Bleeding is not a common feature. However, in this case, the main symptom is recurrent episodes of bleeding from the lesion in the upper labial gingiva, which is located in a port-wine stain (PWS) mucosal and facial region.

The PWS is a congenital facial capillary malformation that occurs in 3 per 1000 live births. Above 40% of all the cases of port-wine stain are limited to the cutaneous distribution of the trigeminal nerve. The pathogenesis of PWS remains controversial [15], and the gingival lesions have been reported to be associated with PWS, particularly in association with trauma, or laser treatment [16]. The association of the gingival mass with this congenital stain has alerted the clinician to the possibility of a vascular hemangioma or pyogenic granuloma. This association is thought to be caused by the arteriovenous anastomosis in the vascular lesion, which leads to the pyogenic granuloma development [17]. The lesion of the present case is situated in the maxillary labial gingiva and is subjected to continuous trauma during mastication and that is why some amount of ulceration and keratotic surface was evident clinically.

Dental examination revealed a missing upper left central incisor, and the presence of an unsatisfactory cantilever bridge, which harbors dental plaque leading to poor oral hygiene in this area. This phenomenon raises the suspicion of a large pyogenic granuloma resulting from plaque accumulation and continuous irritation from the mobile bridge. Incidentally, the panoramic view showed a supernumerary microdont lateral to the midline in the radiolucent area, which then suggests the possibility of presence of an odontogenic cyst like dentigerous cyst.

The differential diagnosis for such presentation includes reactive lesions, such as pyogenic granuloma, peripheral giant cell granuloma, and neoplastic lesions like peripheral ameloblastoma and basal cell carcinoma. Immunohistochemical staining of tissue against Ki67, CD10, and BER-Ep-4 is done to confirm the disease entity and to study the behavior of the lesion.

The histopathological findings demonstrate proliferation strands of the odontogenic epithelium, arranged in many patterns to resemble that seen in intraosseous ameloblastoma that is follicular, plexiform, basal cell, and acanthomatous. The lesion in this case depicted a follicular pattern with acanthomatous changes, while the islands usually exhibit palisading of columnar basal cells and a stellate reticulum is seldom noticeable. Literature reveals documented cases with clear cell differentiation and ghost cells as variants [18]. The histological features of PA shared many features of BCC. Both are characterized by nests of proliferating basal cells intermixed with fibrous stroma. However, PA is negative for BER-Ep4 and positive for BCC. The differentiation of two disease entity by immunohistochemistry is mandatory because the result will modify the surgical procedure, which requires much larger resection in the case of BCC in comparison with PA, which is treated more conservatively.

Ki67 and CD10 are markers for aggressive behavior with high tendency to local recurrence after resection. CD10 is primarily employed in invasion of the extracellular matrix, and positive stromal cells may signify the aggressiveness of the tumor [19]. The strong expression of CD10 in peritumoral stromal cells in the present case indicated that long-term follow-up is mandatory and local recurrence is expected any time after surgical excision of the tumor. Abdel-Aziz and Amin in their study about 22 cases of ameloblastoma [20] suggest that there is a significant relation between Ki labeling index of nuclear proliferation and recurrence of ameloblastoma. The high Ki67 labeling index and the strong expression of CD10 on stromal cells in the present case were the best immunohistochemical markers used for prediction of recurrence in ameloblastoma.

The recommended treatment of choice is conservative supraperiosteal surgical excision [1] together with curettage of the residual bone defects.

In the majority of cases of peripheral ameloblastoma, the lesion is unlikely to present with bone involvement, and this may be due to a barrier created by fibrous tissue of the gingiva and the periosteum. Occasionally, a superficial cupping or saucerization of the cortical plate is seen radiographically or at surgery, and occurrence of cupping or saucerization was thought to be due to tumor pressure rather than direct invasion. In the present case, the tumor was abutting to oral mucosa indicating that the tumor originated from the basal layer of the gingival epithelia. However, the intraoperative findings surprisingly showed multiple scalloping indentations of the underlying alveolar bone with rugged borders, which created some concerns to the nature of the lesion. The surgeon then decided to perform some amount of bone curettage in the region of the bony defect, to ensure complete removal of the tumor.

Although peripheral ameloblastoma is a benign neoplasm with no aggressive behavior or invasive potential, which is different from intraosseous ameloblastoma, some recurrent lesions have been reported with a rate of 19% and one case of metastasis has also been documented [21]. Although adequate disease-free margins are confirmed histologically, long-term follow-up is necessary. In the present case, the behavior of the tumor presenting with recurrent bleeding episodes and the unnatural presence of more extensive bony involvement demand a long-term follow-up.

## 5. Conclusion

Coexistence of port-wine stain with gingival ameloblastoma on the same side has not been reported in the literature. As per our best knowledge, this is the first case of this type. The present case has highlighted the coexistence of both lesions.
